# Flavonoids: A Myth or a Reality for Cancer Therapy?

**DOI:** 10.3390/molecules26123583

**Published:** 2021-06-11

**Authors:** Cinzia Forni, Massimiliano Rossi, Ilaria Borromeo, Giordana Feriotto, Giovambattista Platamone, Claudio Tabolacci, Carlo Mischiati, Simone Beninati

**Affiliations:** 1 Department of Biology, University of Rome Tor Vergata, Via della Ricerca Scientifica, 00133 Rome, Italy; massimiliano87rossi@hotmail.com (M.R.); g.platamone@outlook.it (G.P.); beninati@bio.uniroma2.it (S.B.); 2 PhD Program in Evolutionary Biology and Ecology, Department of Biology, University of Rome Tor Vergata, Via della Ricerca Scientifica, 00133 Rome, Italy; 3 Department of Physics, University of Rome Tor Vergata, Via della Ricerca Scientifica, 00133 Rome, Italy; ilaria18scv@hotmail.it; 4 Department of Chemistry and Pharmaceutical Sciences, University of Ferrara, 44121 Ferrara, Italy; giordana.feriotto@unife.it; 5 Department of Oncology and Molecular Medicine, Istituto Superiore di Sanità, 00161 Rome, Italy; claudiotabolacci@tiscali.it; 6 Department of Neuroscience and Rehabilitation, University of Ferrara, 44121 Ferrara, Italy; msc@unife.it

**Keywords:** flavonoids, cancer, oxidative stress, inflammation, apoptosis/autophagy, metastasis, angiogenesis

## Abstract

Nutraceuticals are biologically active molecules present in foods; they can have beneficial effects on health, but they are not available in large enough quantities to perform this function. Plant metabolites, such as polyphenols, are widely diffused in the plant kingdom, where they play fundamental roles in plant development and interactions with the environment. Among these, flavonoids are of particular interest as they have significant effects on human health. In vitro and/or in vivo studies described flavonoids as essential nutrients for preventing several diseases. They display broad and promising bioactivities to fight cancer, inflammation, bacterial infections, as well as to reduce the severity of neurodegenerative and cardiovascular diseases or diabetes. Therefore, it is not surprising that interest in flavonoids has sharply increased in recent years. More than 23,000 scientific publications on flavonoids have described the potential anticancer activity of these natural molecules in the last decade. Studies, in vitro and in vivo, show that flavonoids exhibit anticancer properties, and many epidemiological studies confirm that dietary intake of flavonoids leads to a reduced risk of cancer. This review provides a glimpse of the mechanisms of action of flavonoids on cancer cells.

## 1. Introduction

The cancer mortality rate has declined over the years due to research and prevention, but its incidence rate has increased. Several studies have highlighted the role of a plant-based diet in the prevention of diseases related to the onset of tumors [1]. The benefits of a plant-based diet could derive from the presence of various bioactive components—such as phenolic compounds, carotenoids, and particularly flavonoids—in vegetables. The latter are considered indispensable and present in various nutraceutical, cosmetic, pharmaceutical, medicinal, and cosmetic applications. Due to these applications, research on flavonoids has increased substantially in recent years.

**Figure 1:**
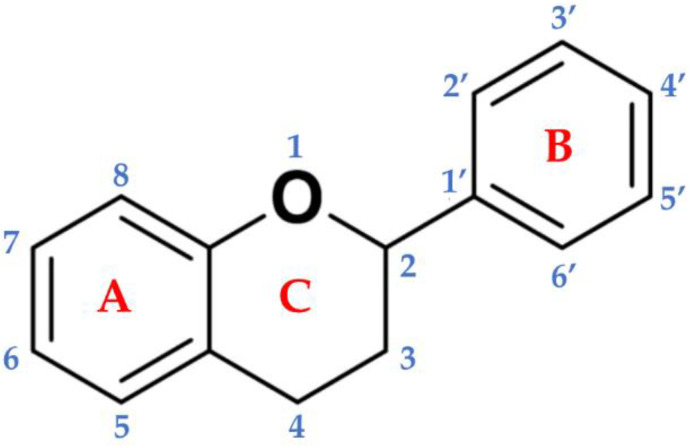
Basic backbone of flavonoids. The chemical structure is composed of two benzene rings (**A** and **B**) linked to the heterocyclic pyranic ring (**C**).

Flavonoids are a subgroup of secondary metabolites belonging to a large collection of phenolic compounds synthesized by plants. They are widely distributed among photosynthetic organisms, and are abundant in foods and beverages of plant origin (Table 1), where qualitative and quantitative compositions can vary considerably. The chemical structure is composed of a skeleton with 15 carbon atoms, containing two benzene rings (A and B) linked to the heterocyclic pyranic ring (C) [2]. Flavonoids can be divided into several subgroups: flavones, flavonols, flavanones, flavanonols, flavanols or catechins, anthocyanins, and chalcones [3]. This distinction is derived from the basic structure of the flavonoid (Figure 1), the flavone ring, which represents the main part of the flavonoid and the degree of unsaturation and oxidation of the carbonaceous ring. Furthermore, in plants, the aglycone is the basic flavonoid structure; however, methyl ethers and acetyl esters of the alcohol group may be present, as well as glycosides formed through linkage with a carbohydrate, such as l-rhamnose, d-glucose, glucose-rhamnose, galactose, or arabinose [4].

Well over 10,000 molecules belong to the large group of flavonoids [12,13]. This number increases considerably if we consider not only the products derived from the flavonoids and formed during the processing and storage of food, but also the metabolites and conjugates produced in the body following their intake. Therefore, the concentrations of flavonoids, and the structural complexity and physicochemical characteristics, vary greatly depending on the source and matrix [14].

It is very difficult to estimate the dietary intake of flavonoids due to their quantitative and qualitative variability in vegetables and fruits, which can hinder the establishment of epidemiologic relationships in regards to their impact on human health and disease. The numerous studies on absorption and bioavailability present in the literature have been reviewed by different authors [15,16,17]. Several factors can affect flavonoids bioavailability, such as molecular weights, glycosylation, and esterification, causing a degree of uncertainty about the real levels of their bioavailability and absorption in the human body [17].

A detailed description of the metabolic conversion of flavonoids following dietary intake is provided by Crozier and colleagues [16] and Landete [18]. Briefly, the metabolic conversion of flavonoids can take place in the small intestine with the release of aglycones as a result of hydrolases activities. This step is followed by the conversion in the liver, where conjugated forms, i.e., *O*-glucuronides, sulfate esters, and *O*-methyl esters of flavonoids are produced. The body can treat these metabolites as xenobiotics; thus, removing them from bloodstream [16,18]. The glucuronides and sulfate derivatives can be more easily excreted via urine and bile [18]. Consequently, the analysis of plasma may not provide valuable information about the profiles of these metabolites, while urinary excretion presents great individual variability depending on the classes of flavonoids and the possibility of metabolites absorption by the body tissues.

Moreover, the compounds, not absorbed by the intestine, will further proceed to the colon, where they will be structurally modified by colonic microflora. The derived catabolites can be absorbed into the bloodstream and finally excreted in urine. Moreover, the flavonoids can modulate gut microbiota composition by increasing the population of beneficial bacteria, e.g., *Bifidobacterium* and *Lactobacillus*, and inhibiting the growth of different pathogens [19]. Such ability of flavonoids provides an important anticolitic mechanism.

### 1.1. Biosynthetic Pathway of Flavonoids in Plants

**Figure 2:**
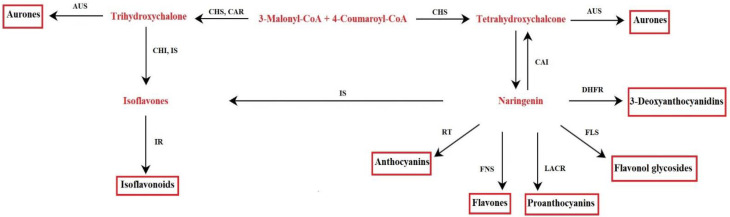
Flavonoid biosynthetic pathway. Aureusidin synthase (AUS), chalcone isomerase (CHI), chalcone reductase (CAR), chalcone synthase (CHS), dihydroflavonol-4-reductase (DHFR), flavonol synthase (FLS), flavone synthase (FNS), isoflavone reductase (IR), isoflavone synthase (IS), leucoanthocyanidin reductase (LACR), rhamnosyl transferase (RT).

The metabolism of flavonoids involves genes already present in the first terrestrial plants, liverworts, and mosses [20]. The biochemical pathway was characterized through the study of mutants with an altered synthesis of flavonoids present in various plant species [21]. The key precursors for flavonoid synthesis are phenylalanine and malonyl-CoA produced by the shikimate pathway and the TCA cycle (tricarboxylic acid cycle). Through the shikimate pathway, aromatic amino acids are produced in plants, bacteria, and fungi. This pathway consists of seven enzymatic reactions, starting from the reaction between phosphoenolpyruvate and erythrose-4-phosphate, up to the synthesis of chorismate, the final product of the pathway, catalyzed by chorismate synthase. The chorismate mutase rearranges the chorismate to prephenate; the latter is the substrate used to synthesize phenylalanine [22]. In plants, phenylalanine is the precursor of 4-coumaroyl-CoA, following phenylalanine ammonia-lyase activity (PAL) and 4-coumarate-CoA ligase. To initiate the synthesis of flavonoids, 4-coumaroyl-CoA reacts with malonyl-CoA [23] (Figure 2). These enzymes localize on the cytosolic side of the endoplasmic reticulum (ER), as suggested by immune localization experiments, and recovered in the soluble fraction of cell extracts. Furthermore, enzymes are associated with each other by protein–protein interactions on the surface of the endoplasmic reticulum (ER); thus, forming a complex [21,24,25]. Data on the co-localization of some enzymes at the tonoplast and the nucleus have suggested a dynamic behavior of the biosynthetic complex. This would favor both the channeling and the displacement of the final products to meet the cells’ physiological needs [24]. The compounds are targeted to vacuoles as storage organelle (i.e., anthocyanins, flavonol, and flavone glycosides) or to cell walls [21]. However, it is essential to point out that under certain physiological conditions, plant cells are able to remobilize flavonoids from vacuole deposits, so transport across the tonoplast is not unidirectional [25]. In addition to vacuoles and cell walls, flavonoids are found in the cytosol, ER, chloroplasts (i.e., quercetin and kaempferol glycosides), nucleus (i.e., isoflavonoids coumestrol and 4’,7-dihydroxyflavone in *Medicago truncatula*), and small vesicles, as well as apoplastic space (i.e., flavone, flavonol aglycones, and isoflavones) [25]. An efficient transport system of flavonoids within cells seems to be the basis of their wide distribution to different cell compartments. Two main systems seem to be involved in the transport of flavonoids, one based on membrane vesicles and the other on a membrane transporter, which does not appear mutually exclusive [25].

### 1.2. Role of Flavonoids in Plants

The conservation of genes involved in the metabolism of flavonoids during the evolution of terrestrial plants is a clear indication of their fundamental role in the physiology of the plant [15]. Flavonoids are responsible for the color and aroma of flowers, are involved in reproductive strategies, protect cells from harmful UV radiation (essential for the life of terrestrial plants), and play a role in disease resistance, as well as in symbiotic association (i.e., as signal molecules in plant-microorganism symbiosis). By being involved in stress responses, they protect the plant from harsh environmental conditions [26,27,28]. The widespread diffusion of flavonoids suggests that their antioxidant activity is a robust feature for the survival and fitness of terrestrial plants. In fact, their synthesis is enhanced after exposure of the plant to severe stress, as their powerful antioxidant activity can counteract the deleterious effects of reactive oxygen species (ROS) [29,30].

### 1.3. Flavonoids and Biotechnology

Flavonoids have been associated with many favorable agronomic traits and health benefits for humans, so their metabolic engineering is an important goal for plant biotechnology [25]. The amounts of flavonoids in plants vary, depending on the species, growing conditions, and stage of development. In fact, even if medicinal and aromatic plants are quite efficient in producing these molecules, the field-grown plants cannot always represent a good source of these metabolites. This is due to the difficulties in plant cultivation, seasonal variations in productivity, tissue/organ-specific production, and problems related to purification. For these reasons, the industrial production of polyphenols would be difficult to sustain if the plants grown in the field were the only source of raw material. On the other hand, the highly complex structures and stereospecificity of flavonoids often make chemical synthesis not economically feasible [31]. In vitro techniques may represent a tool for improving flavonoid biosynthesis and availability throughout the year to overcome these problems. Different plant in vitro cultures (i.e., callus, cell suspension cultures, organ, and hairy root cultures) and transformation techniques have been utilized to investigate and to improve the synthesis of these important molecules [31,32,33,34,35]. Several approaches have been taken into account, such as selecting high-yielding lines, precursor feeding, and the use of elicitors [36]. The latter involves the addition to the culture media of molecules of either biological or chemical synthesis, capable of stimulating the accumulation of secondary metabolites in the plant as a defense response to stress conditions [28], triggered and activated by elicitors [31,32,33,36]. Positive results have been obtained in different species [37], and, in this perspective, the use of elicitors can be considered for future development on an industrial scale.

Furthermore, the improved knowledge on the role of miRNAs in the regulation of the biosynthetic pathway of flavonoids will allow improvements in the metabolism of these molecules. Modulation of miRNA levels could be a powerful tool both for obtaining better yield and for the synthesis of desired combinations of metabolites [38].

## 2. Cancer Preventive Activities of Flavonoids

**Figure 3:**
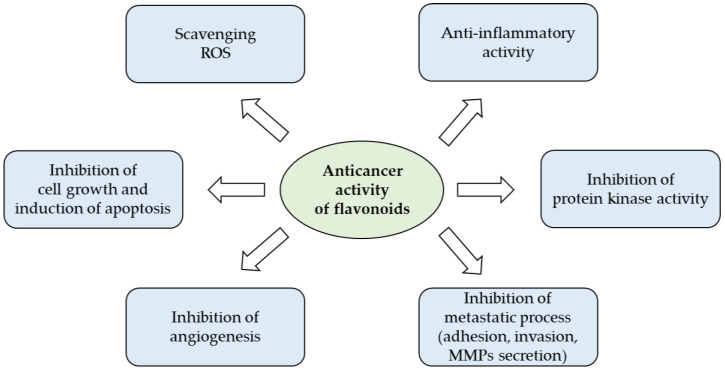
Anticancer potential of flavonoids (from [40] with modifications).

The wide spectrum of biological actions carried out by flavonoids largely depends on their characteristic of being powerful anti-inflammatory and antioxidants that counteract free radicals, linked in an important way for many chronic degenerative diseases (Figure 3). Under pathological conditions, the increase of free radicals damages different types of molecules, such as nucleic acids, proteins, and lipids, and resulting in cell aging and death, but also in the promotion of carcinogenesis [39].

### 2.1. Flavonoids and Chronic Inflammation

Cancer is considered a disease related to chronic inflammation [41]. In various inflammatory diseases, the outcome leads to carcinogenesis. In the biliary tract, cholangiocarcinoma produces a chronic inflammatory infiltrate, due to the infection with *Clonorchis sinensis* [42]. *Helicobacter pylori* represents one of the main causes of adenocarcinoma and lymphoma of the lymphoid tissue associated with the gastric mucosa [43]. Chronic hepatitis B and C virus infection can lead to hepatocellular carcinoma, the third leading cause of cancer death [44]. Finally, papillomavirus infection is a leading cause of penile and anogenital cancer in humans. In addition, the risk of developing bladder cancer may increase following schistosomiasis, as can the risk of contracting Kaposi’s sarcoma following human herpesvirus type 8 infection. Further forms of chronic inflammation, besides those generated by microbial infections, can contribute to carcinogenesis. Increased risk of pancreatic, esophageal, and gallbladder cancer has been described as a consequence of inflammatory diseases such as Barrett’s metaplasia, esophagitis, and chronic pancreatitis [45,46]. Possible associations have also been found between Marjolin ulcer and skin cancer [47], asbestos and mesothelioma [48], cigarette smoke and bronchial cancer [48], chronic asthma and lung cancer [49], ulcerative lichen planus and squamous cell carcinoma [50], foreskin inflammation/phimosis and penile cancer [51], and between pelvic/ovary inflammation and ovarian cancer [52]. Prostate cancer has been associated with chronic prostatitis caused by a persistent bacterial infection or by non-infectious mechanisms [53]. Therefore, the association between chronic inflammation and the development of cancer appears supported by an increasing body of evidence.

In this regard, flavonoids have demonstrated the dual ability to reduce inflammation and the proliferation of tumor cells. Taxifolin, a flavanonol found in conifers, has either anti-inflammatory or antiproliferative effects. In Swiss albino mice challenged with benzopyrene, a mutagen that is frequently present in cigarette smoke and car exhausts, it exerted suppressed inflammation via stimulating the Nrf2 (nuclear factor erythroid 2–related factor 2) signaling pathway, which plays a central role in providing resistance to oxidative stress and inflammation by inhibiting NF-kB [54,55]. Chrysin is an aglycone flavonoid with anti-inflammatory functions. The administration of chrysin in mice challenged with LPS (lipopolysaccharide) reduced the development of lung injuries by suppressing the inositol-requiring enzyme 1α/thioredoxin interaction protein/nucleotide-binding oligomerization domain-like receptor protein 3 pathway [56]. In rats, it prevented the myocardial complications of hypercholesterolemia-triggered oxidative stress through the activation of endothelial nitric oxide synthase and Nrf2 target genes such as SOD (superoxide dismutase) and catalase [57]. Furthermore, chrysin significantly inhibited proliferation and induced apoptosis on human cervical cancer cells [58] and colorectal cancer cells [59] by modulating various apoptotic genes and AKT/MAPK pathway genes. These results highlight two distinct mechanisms through which flavonoids determine the effects on inflammation and cell proliferation: on the one hand, they activate the Nrf2 pathway to inhibit NF-kB and trigger the anti-inflammatory effect; on the other hand, they act on cell proliferation by modulating the genes involved in apoptosis and the AKT/MAPK (protein kinase B/mitogen-activated protein kinase) pathway.

### 2.2. Flavonoids and Oxidative Stress

**Figure 4:**
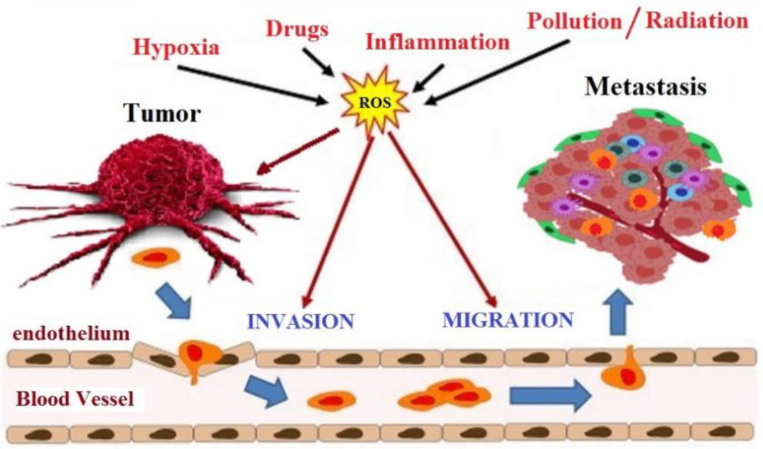
Involvement of oxidative stress in cancer progression.

The intracellular environment in cancer cells has a high level of ROS than the normal cell, principally hydrogen peroxide, due to an antioxidant system that is no longer efficient. In normal cells, adequate glutathione (GSH/GSSG) ratio converts the hydrogen peroxide to water. When the glutathione ratio decreases, the hydrogen peroxide is converted to hydroxyl radical (OH^·^), which is a very reactive radical that leads to DNA damage and mutations in tumor suppressor genes, an initial critical event that triggers carcinogenesis [60]. At least three stages characterize the development of cancer: initiation, promotion, and progression. Oxidative stress is involved in all phases of this process (Figure 4). During the initiation phase, ROS can damage DNA by introducing gene mutations and structural alterations in the DNA. In the promotion phase, ROS have a fundamental role in increasing cell proliferation or reducing cell apoptosis as a consequence of modification of gene expression, communication between cells, and intracellular signaling pathways [61]. Finally, oxidative stress contributes to the progression of the tumor process through further mutagenesis in the initiated cell population [62]. The therapeutic goal of many anticancer drugs is to push up the already high level of ROS present in tumor cells to trigger the apoptosis cascade [63]. Even flavonoids, although recognized for their antioxidant activity, can have pro-oxidant activity and, thus, trigger apoptosis in cancer cells.

Naringenin is a flavanone most abundant in grapefruit, tangerine, orange, raw lemon peel, and raw lime peel. It stopped the cell cycle and induced apoptosis in several human tumor cells [64,65], and also suppressed the invasiveness and metastatic potential of gastric cancer cells and hepatocellular carcinoma cells [66,67]. Naringenin had a pro-oxidant effect as reduced the glutathione reductase, glutathione *S*-transferase and glyoxalase activities in tumor cells, which in turn reduced the mechanisms of detoxification of hydrogen peroxide consenting the accumulation and the augment of lipid peroxidation with consequent cell membrane damage [68]. Interestingly, a recently concluded phase 1 clinical trial has highlighted the safety and pharmacokinetics of naringenin [69]. Naringenin, 4 h after the administration of a single dose of *Citrus sinensis* extract (sweet orange), was detectable in plasma at a concentration of 43 µM.

### 2.3. Flavonoids and Apoptosis/Autophagy

The search for anticancer therapies is currently focused on the induction of apoptosis of cancer cells [70]. Unfortunately, cancer cells are able to avoid the activation of the apoptotic cascade, defending themselves from cell death. Furthermore, tumor development is favored by the induction of drug resistance [71]. The modulation of Bcl-2 and other proteins, allows flavonoids, such as casticin, isolated from the *Vitex agnus-castus* species, widely used in traditional Chinese medicine as an anti-inflammatory agent, to trigger apoptosis by modulating Bcl-2 and other pro-survival. This molecule triggers the intrinsic pathway of apoptosis by downregulating Bcl-2, Bcl-xL, survivin, and upregulating Bax, as evidenced in numerous tumor lines of gallbladder cancer, esophageal cancer, colon cancer, leukemia, and glioblastoma [72]. Similarly, vitexin is a naturally derived flavonoid compound extracted from the Chinese herb *Crataegus pinnatifida* that has been shown to reduce Bcl-2/Bax ratio, release of cytochrome c from mitochondria, and in human non-small cell lung cancer A549 cells, caspase-3 cleavage [73].

Downregulation expression of antiapoptotic molecules such as Bcl-2 and Bcl-xL and up-regulation of the expression of pro-apoptotic molecules, such as caspase-3 and caspase-9, was observed in the inhibition of proliferation of a line of human metastatic ovarian cancer (PA-1) exerted by quercetin [74] one of the most abundant flavonoids in onions and broccoli.

Autophagy is a highly conserved stress-induced catabolic process that positively regulates the cell death process. Several anticancer drugs triggered autophagy and, therefore, its induction represents a potential strategy for cancer therapy [75]. The aqueous extract of Allspice is rich in different types of flavonoids. In breast cancer cells, it activated autophagy, in vitro and in vivo, and induced cell death by suppressing the Akt/mammalian target of the rapamycin (mTOR) pathway [76]. Similarly, in SK-HEP-1 human hepatic cancer cells, kaempferol induced autophagy through Akt signaling and adenosine monophosphate-activated protein kinase (AMPK), and through the downregulation of CDK1/cyclin B led to G2/M arrest [77]. Furthermore, genistein induction of autophagy in multiple types of cancer, such as breast, prostate, and uterus cancer, appears to underlie its anti-tumor effect [78].

### 2.4. Flavonoids Targeting Cancer Stem Cells

Cancer stem cells (CSCs) are a small subpopulation of cells in a tumor that self-renewing and able to initiate and sustain tumor growth. Furthermore, CSCs in cancer play a critical role in the onset, maintenance, progression, drug resistance, and recurrence or metastasis [79]. Accumulating pieces of evidence suggest that dietary phytochemicals, including flavonoids, are promising agents to counteract CSCs [80]. For example, it has been demonstrated that naringenin inhibits breast cancer stem cells through the increase of p53 and estrogen receptor α similarly as found for hesperidin [81].

Apigenin is a common flavone principally found in chamomile, celery, and parsley. The anticancer activity of apigenin has been observed in glioblastoma (the most common primary and aggressive brain tumor). In fact, Kim and colleagues [82] demonstrated that apigenin (and quercetin) is able to interfere with the self-renewal capacity and invasiveness of glioblastoma stem-like cells through the downregulation of c-Met signaling pathway. Apigenin increases the antineoplastic activity of cisplatin in CD44+ prostate cancer stem cell populations [83] and suppresses the stem cell-like properties and tumorigenic potential of triple-negative breast cancer cells [84]. The inhibition of self-renewal ability and the restoration of radio-sensitivity have been demonstrated in oral cancer stem cells for luteolin [85], a flavone found in a large variety of dietary source including celery, carrots, peppers, olive oil, rosemary, and oregano. The flavonol quercetin is a molecule of medical interest, as it possesses anticancer potential [86]. In fact, quercetin targets several types of CSCs, including pancreatic [87], breast [88], and gastric [89] stem cells.

### 2.5. Anti-Angiogenic and Anti-Metastatic Properties of Flavonoids

Flavonoids play an interesting role as inhibitors of angiogenesis. Angiogenesis consists in the development of new blood vessels, which is a process fundamental for tissue growth, wound healing, and embryonic development, but it represents a negative feature in the presence of a tumor as more blood vessels carry more nutrients to the cancer cells allowing them to better live and proliferate. It is a process tightly controlled by a wide range of inducers, such as vascular endothelial growth factor (VEGF) and adhesion molecules, as well as by various inhibitors including angiostatin and thrombospondin, and stimulated by many factors contributing to inflammation and cancer, therefore indicating that angiogenesis, inflammation, and cancer are closely related processes [90]. In recent years, the development of angiogenesis inhibitors has been a hot spot of anticancer research as this uncontrolled process is a fundamental step in cancer growth, invasion, and metastasis. Following this effort, FDA approved the use of numerous anti-angiogenesis drugs for cancer treatment [91]. New molecules capable of inhibiting tumor angiogenesis are being tested. Wogonin, an *O*-methylated flavone, a flavonoid-like chemical compound synthetized by *Scutellaria baicalensis,* inhibits LPS-induced angiogenesis both in vitro and in vivo [92]. Genistein inhibits angiogenesis by modulating the expression of VEGF, metalloproteases (MMP) and epidermal growth factor receptor (EGFR) [93]. In the endothelial cells of the human umbilical vein, stimulated by VEGF (HUVECs), Kaempferol inhibits angiogenesis by acting on the VEGF receptor 2. This process is also carried out thanks to the down-regulation of P13kt/Akt together with the mitogen-activated protein kinase (MEK) and the ERK pathways [94].

Luteolin (8-*C*-β-d-glucopyranoside), a glycosyl dietary flavonoid, reduces tumor invasion, into 12-*O*-tetradecanoylphorbol-13-acetate (TPA)-treated MCF-7 breast cancer cells, blocking expression of MMP-9 metalloproteinase and interleukin-8 (IL-8) [95]. In gastric cancer cells, quercetin showed antimetastatic effects via breakdown of urokinase plasminogen activator (uPA)/uPA receptor (uPAR) function, by modulating NF-κB, PKC-δ, ERK1/2, and AMPK [96]. Recently, Yao et al. reported that in A375 human melanoma cells, luteolin inhibits proliferation, migration, and invasion by inducing dose-dependent apoptosis. In the same cell model, inhibition of Akt and PI3K phosphorylation was also observed. The same authors have collected experimental evidence that luteolin allows the overexpression of tissue inhibitors of the metalloproteinase (TIMP)-1 and TIMP-2 and reduces the expression of MMP-2 and MMP-9 [97]. Further experimental results highlighted that luteolin significantly reduced tumor growth of A375 cells in a mouse xenograft model, confirming that the antitumor activity is derived from down-regulation of MMP-2 and MMP-9 expression through the PI3K/Akt pathway [97].

### 2.6. Flavonoids and Cancer Cell Differentiation

Differentiation therapy aims to induce the differentiation of cancer cells; thus, reducing their proliferation [68]. Differentiation therapy compared to conventional chemotherapy has the advantage of being less toxic and, therefore, causing fewer side effects to the patient [98]. Quercetin and pelargonidin induce differentiation on highly metastatic B16-F10 melanoma murine cells by a mechanism involving transglutaminase type 2 [99]. All-trans retinoic acid (ATRA) has wide clinical use in differentiation therapy on patients with acute promyelocytic leukemia (APL). However, prolonged treatment results in drug resistance and requires an increasingly higher dosage [100]. The emergence of drug resistance phenomena needs the development of new agents with greater differentiation induction activity. Flavonoids have interesting characteristics in this sense. In fact, they are able to induce cellular differentiation of APL cells. However, flavone structure might be crucial for the induction of cell differentiation. Indeed, in APL cells, quercetin induces their differentiation into monocytes and apigenin and luteolin induce their differentiation into granulocytes. On the contrary, galangin, kaempferol, and naringenin did not induce any differentiation in APL cells [100].

Recently, Moradzadeh et al. [101] reported that epigallocatechin gallate (EGCG), a green tea polyphenol, in granulocyte differentiation of APL HL-60 and NB4 cells, possesses a similar effect to ATRA. In both of these cell lines EGCG, reduced the expression of histone deacetylase 1. Furthermore, in NB4 cells, EGCG also reduced the expression of a relevant clinical marker PML-RARα. Cell differentiation was induced by wogonin, in the K562 cell line, a primary chronic myeloid leukemia (CML) cell model. The same result was observed in patient-derived primary CML that was sensitive and resistant to imatinib. Upregulation of the transcription factor GATA-1 and increased binding between GATA-1 and the transcriptional coactivator FOG-1 was also observed in these cells [102]. Several observations provide evidence to support the potential application of flavonoids in the treatment of patients with different types of cancer. In tumor cells isolated from various solid tumors, such as malignant melanoma, breast cancer, glioma, and hepatoma, flavonoids treatment-induced differentiation has been demonstrated [103]. Specifically in breast cancer stem cells, cell differentiation induced by genistein [78,93] and a flavonoid isolated from licorice (*Glycyrrhiza* sp.), isoliquiritigenin, has been observed [104].

In the treatment of APL NB4 cells, with dihydromyricetin (DMY), a dihydroflavonol extracted from *Ampelopsis* sp., it was observed that this synergized with ATRA, to promote cell differentiation [105]. ATRA-induced phosphorylation of p38 MAPKs activates STAT1, and STAT1 plays a key role in the terminal differentiation of myeloid cells through the regulation of cell cycle proteins and specific myeloid transcription factors. DMY-enhanced differentiation, when combined with ATRA was dependent on the increased activation of p38MAPK/STAT1 signaling pathway. Interestingly, DMY alone was unable to activate differentiation and reduced the phosphorylation of p38 MAPK with a consequent reduction in STAT1 activity [105]. This unexpectedly different behavior, in the activation of the pathway, suggests that it is not possible to predict the biological effect derived from the combination of a generic flavonoid with a conventional drug simply basing on the knowledge of their mechanism of action studied in single treatments, as it may not be the same. Therefore, all flavonoids could be possible differentiation enhancers in combination with conventional drugs.

### 2.7. Flavonoids to Improve Sensitivity to Chemotherapy

**Figure 5:**
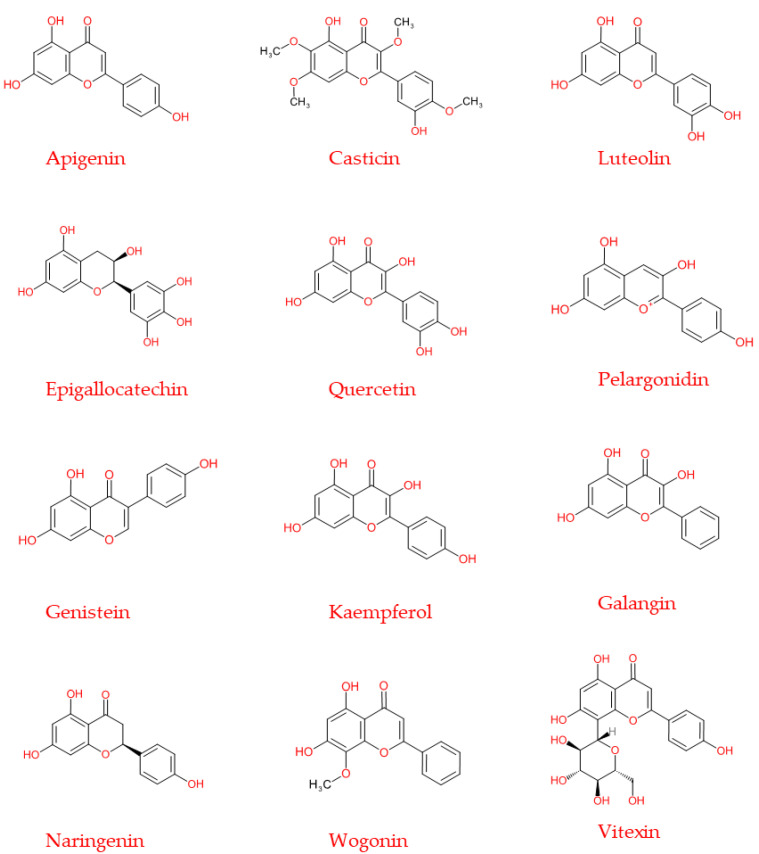
Chemical structure of the principal flavonoids discussed in the present review, also used in the experimental chemotherapy treatments.

Combined treatments with multiple molecules can improve the overall clinical efficacy of current anticancer drugs [68,106]. Due to multi-drug resistance and tumor recurrence, the development of new strategies to improve sensitivity to chemotherapy and minimize adverse side effects is still urgent. In this regard, flavonoids have been considered promising candidates by virtue of their anticancer activity (Figure 5). Yuan et al. [107] provided evidence of the antiproliferative efficacy of the combination of arsenite and delphinidin (the latter being one of the anthocyanin compounds) on human NB4 and HL-60 APL cells. Delphinidin sensitized arsenite-resistant leukemia cells to apoptosis modulating the amount of glutathione and reducing the activity of NF-κB. They also showed that the combined treatment was selective as it increased the cytotoxicity of arsenite against cancer cells but not on human peripheral blood mononuclear cells [107].

Furthermore, combined treatment with flavonoids exerted beneficial effects in various cell types stabilized from solid tumors. Quercetin has been demonstrated to sensitize human glioblastoma U87 and U251 cells to temozolomide, an oral alkylating chemotherapeutic agent, in vitro via inhibition of heat-shock protein 27 [108]. Flavonoids are able to enter the brain [109]. The anticancer potential of a combination of isoflavone biochanin A and temozolomide against glioblastoma U87 and T98G cells was associated with enhanced expression of p-p53, inhibition of cell viability and expression of cell survival proteins EGFR, p-Akt, p-ERK, membrane-type-MMP1, and c-myc [110]. Combined treatment in cancer cells induced cell cycle arrest in the G1 phase and a substantial change in energy metabolism from anaerobic to aerobic [95]. In colon cancer cells, casticin potentiated the apoptosis induced by TNF-related apoptosis-inducing ligand (TRAIL) through upregulation of death receptor 5 and downregulation of survival proteins, such as survivin, Bcl-xL, Bcl-2, cellular FLICE-like inhibitory protein (cFLIP), and X-linked inhibitor of apoptosis protein (XIAP) [95]. In human colorectal adenocarcinoma LoVo cells, Palko-Łabuz et al. recently demonstrated that the flavonoid baicalein potentiates the anti-proliferative and pro-apoptotic effect of statins, making doxorubicin treatment effective in an otherwise resistant cell line [111]. In addition, green tea EGCG catechin suppresses tumor growth and increases the therapeutic efficacy of drugs in various cancers, such as that of 5-fluorouracil (5-FU) on colon cancer cells by inhibiting glucose-regulated protein 78 (GRP78)/NF-κB/miR-155-5p/MDR1 pathway [112].

It has been suggested that EGCG polyphenol in tea has the potential to be a therapeutic adjuvant against human metastatic breast cancer [113]. A clinical study showed that breast cancer patients subjected to radiotherapy and oral administration of EGCG exhibited reduced activation of MMP-9/MMP-2 accompanied with low serum levels of VEGF and hepatocyte growth factor (HGF) [113]. In an MDA-MB-231 human breast cancer cell line, luteolin augments the action of doxorubicin and paclitaxel by suppressing Nrf2-mediated signaling and blocking STAT3 [95,114]. Similar activity was observed for the flavonoid glabridin in breast cancer cell lines, MDA-MB-231/MDR1 resistant (with overexpression of P-gp), and in MCF-7/ADR cells (with overexpression of P-gp and MRP2). The sensitizing effect of glabridin may be due to its ability to increase doxorubicin accumulation in MDA-MB-231/MDR1 cells by suppressing P-gp expression and competitively inhibiting the P-gp efflux pump, thereby enhancing the apoptotic activity of doxorubicin [115]. Kundur et al. have shown that quercetin and curcumin administered together have a synergistic antitumor effect on triple-negative breast cancer (TNBC) cells, including the MDA-MB-231 line, enhancing breast cancer type 1 susceptibility protein expression [116].

Recently, Moon and colleagues reported that treatment with nobiletin increased the accumulation of intracellular Adriamycin (ADR) in the human NSCLC A549/ADR cell line by promoting treatment efficacy through a mechanism accompanied by downregulation of the expression of Akt, neuroblastoma-derived MYC (MYCN), GSK-3β, MRP1, and β-catenin [117]. Moreover, in EGFR mutant-resistant NSCLC cells, apigenin coupled with the EGFR tyrosine kinase inhibitor gefitinib inhibited important oncogenic factors such as c-Myc, hypoxia-inducible factor 1 alpha (HIF-1α) and EGFR, and also reduced the use of glucose by suppressing the expression of its transporter, suggesting the possible use of the combination of the two molecules in clinical practice [118]. Activation of the intrinsic apoptosis pathway by G_1_ phase arrest and phosphatase expression increased the cytotoxicity of paclitaxel in prostate cancer cells treated with a citrus-derived polyphenolic flavonoid, naringenin. One of the key negative regulators of the PI3K/Akt signaling pathway, the tensin homolog deleted on chromosome 10 (PTEN), is also involved in this mechanism, along with down-regulation of NF-κB, Snail, Twist, and c-Myc mRNA expression and suppression of cell migration [119]. These results on the combined use of the two molecules in vitro highlight their therapeutic potential in prostate cancer, although detailed evaluation of the mechanism underlying the combined action in vivo is obviously also necessary.

## 3. Conclusions

Flavonoids have shown particularly effective properties in counteracting tumor growth and in making cancer cells resistant to conventional therapies. With the present compilation of information from the current literature, an attempt has been made to highlight the potential of flavonoids in cancer therapy, whether used alone or in combination with chemotherapeutic agents. Although the potential efficacy of flavonoids in counteracting tumor growth has been highlighted, the search for mechanisms of action will still take a long time.

## Figures and Tables

**Table 1:** Main classes of flavonoids in crop species and their main characteristics.

Classes	Representative Flavonoids	Food Sources	Functions	Information	References
**3-Deoxyanthocyanidins**	Apigeninidin, luteolinidin	Sorghum, purple corn	Pigmentation, antioxidant	Anthocyanidins without hydroxyl in carbon 3 of C ring	[5]
**Anthocyanins**	Cyanidin-3-*O*-glucoside, peonidin-3-*O*-glucoside	Blackberry, blueberry, cherry, strawberry	UV protection, pollinators and seed disperser attraction	3-glycoside form of anthocyanidins	[6]
**Flavones**	Apigenin, luteolin	Celery, green peppers, parsley, peppermint, thyme	Natural pesticides in plants, nodulation, UV protection	Double bond between C2 and C3, no substitution in C3 position	[7]
**Flavonols**	Kaempferol, quercetin, myricetin, rutin	Apple, blueberries, broccoli, cabbage, cherries, garlic, onion, tea, red wine	Pigmentation, UV protection, male fertility, signaling	Double bond between C2 and C3, ketone group on C4, hydroxyl on C3 can be glycosylated	[8]
**Isoflavonoids**	Daidzein, genistein, glycitein	Legumes	Nodulation, defense	3-phenylchromen-4-one backbone	[9]
**Proanthocyanidins**	Condensed Tannins	Cocoa beans, apples, red wine	Pigmentation, pathogens and predator defense	Oligomeric flavonoids (catechin and epicatechin oligomers)	[10,11]
